# Effect of Boron Doping on Cellular Discontinuous Precipitation for Age-Hardenable Cu–Ti Alloys

**DOI:** 10.3390/ma8063467

**Published:** 2015-06-11

**Authors:** Satoshi Semboshi, Jun Ikeda, Akihiro Iwase, Takayuki Takasugi, Shigeru Suzuki

**Affiliations:** 1 Kansai Center, Institute for Materials Research, Tohoku University, Gakuen-cho 1-1, Naka-ku, Sakai, Osaka 599-8531, Japan; 2 Department of Materials Science, Osaka Prefecture University, Gakuen-cho 1-1, Naka-ku, Sakai, Osaka 599-8531, Japan; E-Mails: st110005@edu.osakafu-u.ac.jp (J.I.); iwase@mtr.osakafu-u.ac.jp (A.I.); takasugi@mtr.osakafu-u.ac.jp (T.T.); 3 Institute of Multidisciplinary Research for Advanced Materials, Tohoku University, Katahira 2-1-1, Aoba-ku, Sendai 9577-8531, Japan; E-Mail: ssuzuki@tagen.tohoku.ac.jp

**Keywords:** Cu–Ti alloy, boron, discontinuous precipitate, aging, strengthening

## Abstract

The effects of boron doping on the microstructural evolution and mechanical and electrical properties of age-hardenable Cu–4Ti (at.%) alloys are investigated. In the quenched Cu–4Ti–0.03B (at.%) alloy, elemental B (boron) is preferentially segregated at the grain boundaries of the supersaturated solid-solution phase. The aging behavior of the B-doped alloy is mostly similar to that of conventional age-hardenable Cu–Ti alloys. In the early stage of aging at 450 °C, metastable β′-Cu_4_Ti with fine needle-shaped precipitates continuously form in the matrix phase. Cellular discontinuous precipitates composed of the stable β-Cu_4_Ti and solid-solution laminates are then formed and grown at the grain boundaries. However, the volume fraction of the discontinuous precipitates is lower in the Cu–4Ti–0.03B alloy than the Cu–4Ti alloy, particularly in the over-aging period of 72–120 h. The suppression of the formation of discontinuous precipitates eventually results in improvement of the hardness and tensile strength. It should be noted that minor B doping of Cu–Ti alloys also effectively enhances the elongation to fracture, which should be attributed to segregation of B at the grain boundaries.

## 1. Introduction

Age-hardenable Cu–Ti alloys have gained considerable attention for application in electrical devices such as micro-connecters and relay controls because of their excellent mechanical strength, stiffness, stress-relaxation, and good electrical conductivity. Furthermore, owing to the demand for innovative developments in electrical and electric products, the improvements that can be attained in the mechanical and electrical properties of Cu–Ti alloys offer significant advantages. As a result, many studies on fundamental metallurgy as well as practical applications of the alloys have been reported in recent years [1,2,3,4,5,6].

Conventionally, age-hardenable Cu–Ti alloys, which contain approximately 3–6 at.% Ti, are manufactured commercially through the procedures of solid-solution heat treatment at temperatures above 850 °C and then aging between 400 °C and 500 °C. In the initial stage of aging, the supersaturated solid solution of copper, which has a face-centered cubic (fcc) structure, begins to decompose catastrophically into two disordered fcc phases, forming a Ti-depleted region and a Ti-rich region [1,2,7,8,9,10]. The disordered Ti-rich region then becomes ordered, and continuously transforms to fine, needle-shaped, metastable, and coherent precipitates, which are denoted as β′-Cu_4_Ti and have a tetragonal structure (prototype: Ni_4_Mo; space group: *I*4/m; lattice parameters: *a* = 0.583 nm, *c* = 0.362 nm) in the matrix phase. During further aging, coarse, cellular, and discontinuous precipitates, which are composed of the stable intermetallic phase and the terminal copper solid-solution, nucleate and grow in the grain boundaries, consuming the finely dispersed, continuous precipitates of β′-Cu_4_Ti particles [9,10,11,12]. The stable phase has generally been denoted as β-Cu_4_Ti, and it has an ordered orthorhombic structure (prototype: Au_4_Zr; space group: *P*nma; *a* = 0.453 nm, *b* = 0.434 nm, *c* = 1.293 nm). The fine dispersion of fine continuous precipitates (β′-Cu_4_Ti) contributes to the excellent mechanical properties of age-hardenable Cu–Ti alloys. On the other hand, the development of coarse, cellular, discontinuous precipitates (β-Cu_4_Ti and Cu laminates) is unfavorable because they hardly contribute to strengthening owing to the excessive size of several micrometers, and they act as crack-initiation sites owing to the brittleness of β-Cu_4_Ti laminates. In addition, the replacement of favorable fine β′-Cu_4_Ti precipitates with coarse, cellular, discontinuous precipitates is a critical problem that leads to a serious decrease in strength in the later stage of aging. Therefore, the formation of cellular discontinuous precipitates should be suppressed in order to improve the mechanical properties and reliability of the age-hardenable Cu–Ti alloys.

Since grain boundaries act as pipe-diffusion passes for the constituent elements and as the nucleation sites of discontinuous precipitates, we believe that modification of grain boundaries in the Cu–Ti alloys before aging must affect the formation of these discontinuous precipitates. Boron (B) doping should be applied to modify the grain boundaries because B can be easily segregated at the grain boundaries of Cu [8]. There are some reports in the literature on the addition of B to the Cu–Ti alloy system: Sobhani *et al.* showed that large particles of TiB_2_ were located at grain boundaries in the Cu–1Ti–1TiB_2_ (wt.%) alloy, which was prepared by melting [13]; Furuta *et al.* demonstrated that the Cu–4Ti (at.%) alloys containing 0.2–1.0 at.% B, in which TiB_2_ particles were dispersed, had a smaller amount of cellular discontinuous precipitates than conventional Cu–Ti alloys [14]. These reports imply that the addition of B can suppress the formation of discontinuous precipitates in the age-hardenable Cu–Ti alloys, although the effect of B addition is yet to be explained. In the above-mentioned reports, B in the Cu–Ti alloys must be distributed in the matrix as a solute element as well as in the TiB_2_ compound. Thus, it is difficult to examine the contribution of each distribution of B. The purpose of this study is to investigate the effects of the distribution of elemental B in the matrix on the microstructural evolution during aging. To achieve this goal, we prepared Cu–4Ti (at.%) alloys doped with a small amount of B, in which formation of TiB_2_ did not occur because of the low B concentration. We directly investigated the distribution of B in the as-quenched alloy using a time-of-flight secondary-ion mass spectrometer (ToF-SIMS), and thereby evaluated the effect of B doping on the formation behavior of cellular discontinuous precipitates during aging using a field-emission scanning electron microscope (FE-SEM). In addition, the variations in the hardness, tensile strength, and electrical conductivity were also measured and examined on the basis of the precipitation behavior.

## 2. Results and Discussion

### 2.1. Microstructure 

**Figure 1:**
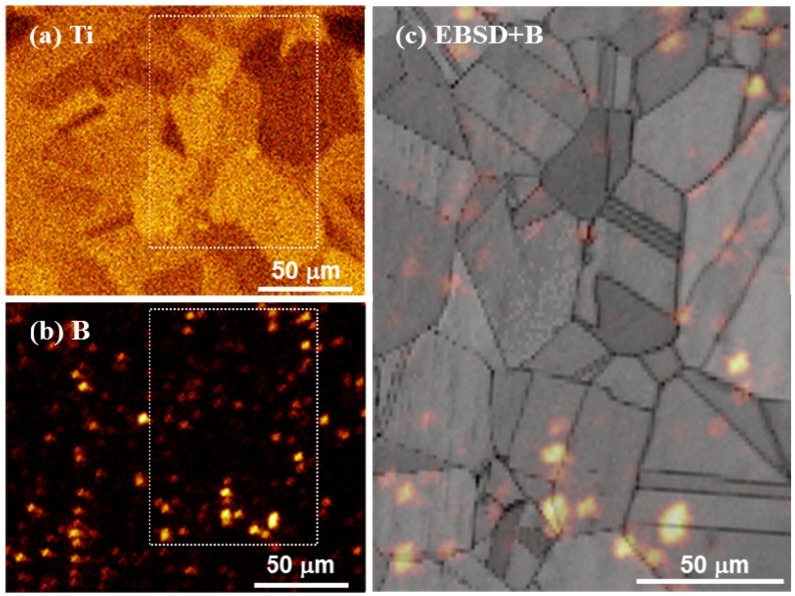
Elemental mapping of (**a**) Ti and (**b**) B in the solid-solution-treated Cu–4Ti–0.03B alloy, obtained by ToF-SIMS analysis. (**c**) EBSD-pattern quality map superimposed on the B map by ToF-SIMS analysis, (**b**). Note that the image in (**c**) corresponds to the region marked by the dotted rectangle in (**a**) and (**b**).

Figure 1 shows elemental Ti and B mappings as well as an elemental B mapping superimposed on a microstructural image obtained by electron backscatter diffraction (EBSD) for the as-quenched Cu–4Ti–0.03B alloy. Our FE-SEM observations revealed only grains of the supersaturated solid-solution with a size of approximately 30 µm, and there was no contrast from a secondary phase. Figure 1a shows that elemental Ti was apparently homogeneously distributed in the alloy, while Figure 1b reveals that elemental B was scattered. Figure 1c indicates that the scattered elemental B was preferentially located at the grain boundaries. It was estimated that 70% of all contrasts from B condensed clusters were located at grain boundaries. We also observed the microstructure of the as-quenched Cu–4Ti alloy, which was prepared by a procedure similar to that used for the Cu–4Ti–0.03B alloy, and we confirmed that the alloy consisted of a solid-solution single phase with a grain size of 30 µm.

**Figure 2:**
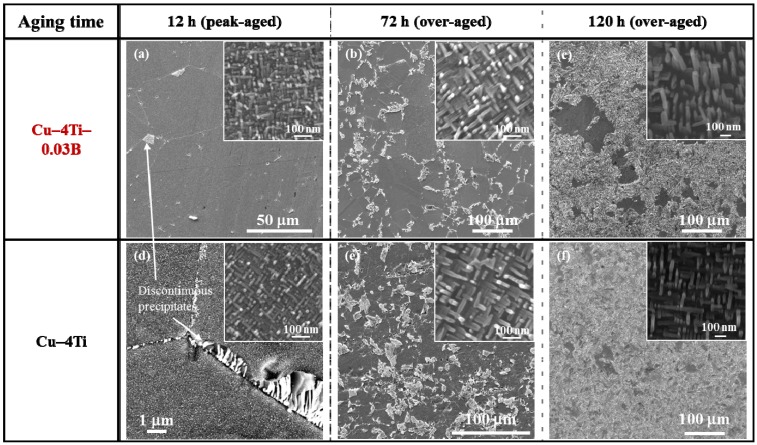
FE-SEM images of Cu–4Ti–0.03B and Cu–4Ti alloys that were aged at 450 °C for 12, 72, and 120 h. The insets are higher-magnification FE-SEM images, which focus on the continuous precipitates.

Figure 2 shows FE-SEM images of the Cu–4Ti–0.03B and Cu–4Ti alloys that were aged at 450 °C for 12, 72, and 120 h. In the image of the Cu–4Ti–0.03B alloy that was aged for 12 h (Figure 2a), we can see fine needle-shaped continuous precipitates of β′-Cu_4_Ti with a length of 30 nm in the grains (inset) and a small amount of cellular discontinuous precipitates at the grain boundaries, as marked by the white arrow. The continuous precipitates grew gradually during aging, but they were consumed as the discontinuous precipitates developed (insets of Figure 2a–c). The microstructural evolution of the Cu–4Ti–0.03B alloy specimens was qualitatively the same as that of the Cu–4Ti alloy specimens without B (Figure 2d–f). When analyzing the volume fraction of the cellular discontinuous precipitates from the FE-SEM images, it was found that the volume fraction in the Cu–4Ti–0.03B alloy specimens was 10%–18% lower than that in the Cu–4 Ti alloy specimens for the aging period between 72 and 120 h. This suggests that the growth of cellular discontinuous precipitates was slower as a result of B doping. 

**Figure 3:**
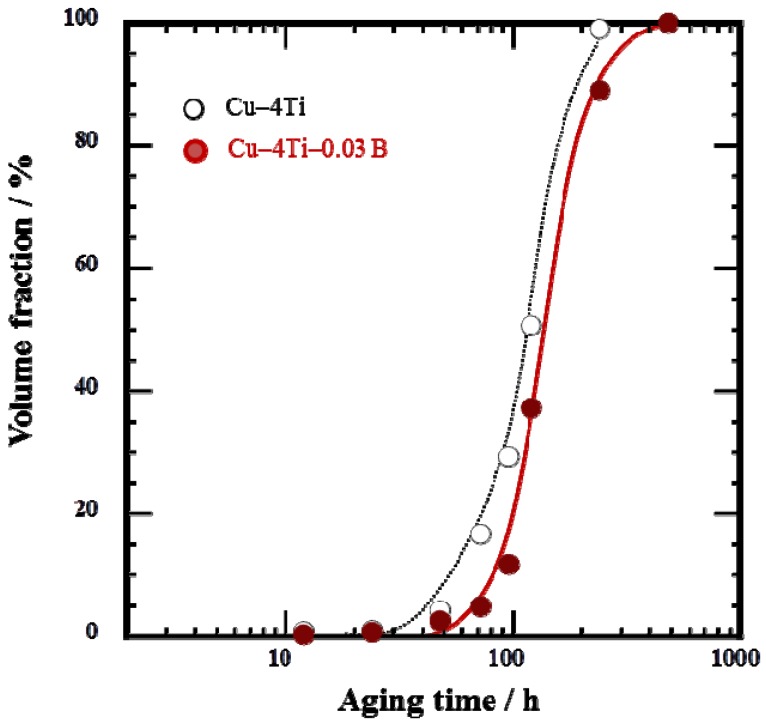
Volume fraction of cellular discontinuous precipitates in Cu–4Ti–0.03B and Cu–4Ti alloys that were aged at 450 °C.

The cellular discontinuous precipitates dominated the entire microstructure of the Cu–4Ti–0.03B alloy specimens that were aged for 480 h, as shown in Figure 3. To investigate whether B was present in the solid-solution or β-Cu_4_Ti phase of the alloys, the two phases were separated by extraction [10,15], after which their chemical composition was analyzed by a technique based on inductively coupled plasma atomic emission spectrometry (ICP-AES). The results, shown in Table 1, reveal that almost all of the elemental B condensed in the solid-solution phase. The amount of elemental B was small in the β-Cu_4_Ti phase, probably because it was difficult to incorporate B into the β-Cu_4_Ti phase during growth. It should be noted that the Ti content of the β-Cu_4_Ti phase in the Cu–4Ti–0.03B alloy specimens exceeded the stoichiometric proportion of 20%, as listed in Table 1. According to the same analysis for the Cu–4Ti alloy specimens, the Ti content of the β-Cu_4_Ti phase was reported to be 20.5 (at.%) [10]. The Ti content in excess of the stoichiometric quantity may have naturally resulted from the phase or it could have been within the analytical accuracy of our study; we will discuss the phenomenon in a future paper.

Elemental B was preferentially located near the grain boundaries in the quenched alloy (Figure 1). It must have originated from B enrichment during solidification, which would have had difficulty diffusing through the alloy during the homogenization and solution-treatment, or it could have been easily trapped by the grain boundaries. Here, we cannot clearly determine whether elemental B existed in the solid-solution or in the fine TiB_2_ particles, although the latter was not detected by FE-SEM observations. During aging, the elemental B should have condensed in front of the growing tips of the β-Cu_4_Ti laminates in the cellular structure; this hypothesis is supported by the growth of β-Cu_4_Ti laminates without incorporation of elemental B, as shown in Table 1. The development of the discontinuous precipitates, which were formed by a reaction at the grain boundaries, was suppressed in the alloys containing B, as shown in Figure 3. This was possibly because the pipe-diffusion of elemental Ti through the grain boundaries was diminished by elemental B located there, or because the mobility of the transformation in front of the grain boundaries was reduced probably owing to the pinning effect of B segregates or secondary-phase particles. The actual mechanism by which B doping of Cu–Ti alloys suppresses the development of discontinuous precipitates must be investigated in further research.

**Table 1:** Chemical composition of Cu solid-solution and precipitation phases in the Cu–4Ti–0.03B alloy that was aged at 450 °C for 480 h. The extraction method and inductively coupled plasma atomic emission spectrometry (ICP-AES) measurements were used to analyze the chemical composition.

	Filtrate	Residue
	(Cu solid-solution)	(precipitates)
**Ti content/at.%**	0.6 ± 0.1	21.5 ± 0.4
**B content/ppm**	393 ± 5	32 ± 3

### 2.2. Mechanical and Electrical Properties

**Figure 4:**
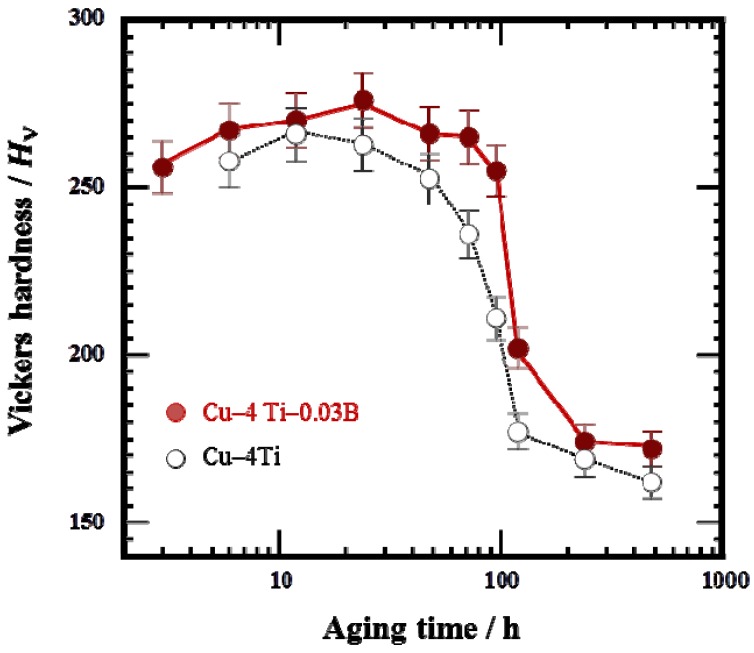
Vickers hardness of Cu–4Ti–0.03B and Cu–4Ti alloys that were aged at 450 °C.

Figure 4a shows the variation in Vickers hardness of the Cu–4Ti–0.03B and Cu–4Ti alloys as a function of aging time. The hardness values of the quenched Cu–4Ti–0.03B and Cu–4Ti alloys were 118 and 120 Hv, respectively, which can be considered the same within experimental accuracy. This suggests that the solid-solution of B, in which the B content was approximately 300 ppm, contributed little to alloy hardening. The hardness of the Cu–4Ti–0.03B alloy specimens increased with aging time and reached a maximum of >270 Hv after 12–24 h, which agree with the results obtained for the Cu–4Ti alloy specimens. After that, the hardness gradually decreased, but the decline was not as significant as that shown by the Cu–4Ti alloy specimens. After aging for 240 h, the hardness decreased to 170 Hv for both Cu–4Ti–0.03B and Cu–4Ti alloy specimens. It can thus be concluded that the hardness of the B-doped alloy specimens was 20–50 Hv higher than that of the alloy specimens without B in the aging period of 72–120 h.

**Figure 5:**
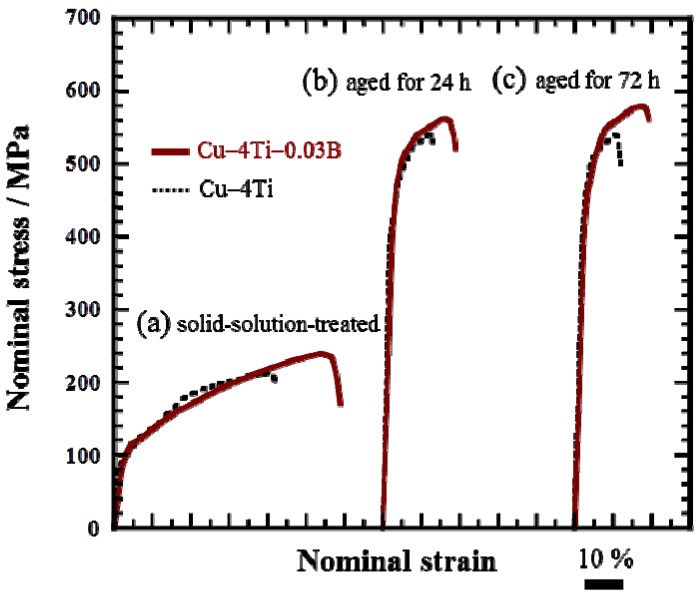
Stress–strain curves of Cu–4Ti–0.03B and Cu–4Ti alloys: (**a**) solid-solution-treated (as-quenched) and aged at 450 °C for (**b**) 24 and (**c**) 72 h.

Figure 5 shows the nominal stress and strain curves of the Cu–4Ti–0.03B and Cu–4Ti alloys in the as-quenched state and after aging at 450 °C for 24 and 72 h, respectively. In both the quenched and aged alloys, the 0.2% proof stress of the Cu–4Ti–0.03B alloy specimens was of the same as that of the Cu–4Ti alloy specimens. On the other hand, the ultimate tensile strength (UTS) of the Cu–4Ti–0.03B alloy specimens was approximately 50 MPa higher than that of the Cu–4Ti alloy specimens in the quenched and aged states. It should be noted that there was no difference between the UTS values of the peak-aged (24 h) and over-aged (72 h) Cu–4Ti–0.03B alloy specimens, which is consistent with the results of Vickers hardness testing. As mentioned earlier, there was no significant difference between the grain sizes of the Cu–4Ti alloy specimens with and without B doping. The distribution of fine β′-Cu_4_Ti precipitates also appeared to be similar in both specimens (see insets of Figure 2c,f). Therefore, the increase in hardness and UTS of the Cu–4Ti–0.03B alloy specimens that were over-aged (aging time: 72 and 120 h) can be explained in terms of the volume fraction of discontinuous precipitates, which was 10%–18% lower than that in the Cu–4Ti alloy specimens for this aging period. The suppression of the formation of discontinuous precipitates resulted in the survival of a large amount of fine continuous precipitates of β′-Cu_4_Ti, which are the primary contributor to the precipitate-induced strengthening of Cu–Ti alloys.

**Figure 6:**
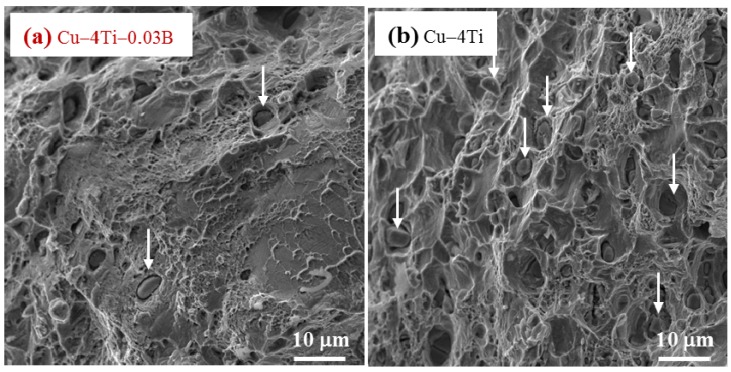
FE-SEM images of the fracture surfaces of (**a**) Cu–4Ti–0.03B and (**b**) Cu–4 Ti alloys that were aged at 450 °C for 72 h. The arrows in (**a**) and (**b**) indicate representative β-Cu_4_Ti inclusions.

It is interesting that the elongation to fracture for all the Cu–4Ti–0.03B alloy specimens quenched and aged for 24 and 72 h was higher by approximately 7% than that for the Cu–4Ti alloy specimens, as shown in Figure 5. Figure 6 shows images of fractures caused by the tensile tests on the Cu–4Ti–0.03B and Cu–4Ti alloys that were aged for 72 h. We can see mostly typical transgranular ductile fractures with patterns of ripples and dimples in the Cu–4Ti–0.03B alloy. The fractrograph of the Cu–4Ti alloy also shows a ductile, but partly brittle, dimple-like fracture surface, which was probably caused by intergranular cracks associated with β-Cu_4_Ti laminates, as marked by the arrows; the intergranular cracks appeared to be more significant in the Cu–4Ti alloy than the Cu–4Ti–0.03B alloy. The improvement in the ductility of B-doped Cu–Ti alloy specimens may also be attributed to the segregation of B at the grain boundaries and suppression of the discontinuous precipitates: the former possibly led to strengthening at the grain boundaries, which would have furthered transgranular plastic deformation prior to intergranular cracking. The latter would have also led to a decrease in the initiation sites of cracking in the brittle β-Cu_4_Ti intermetallic phase.

**Figure 7:**
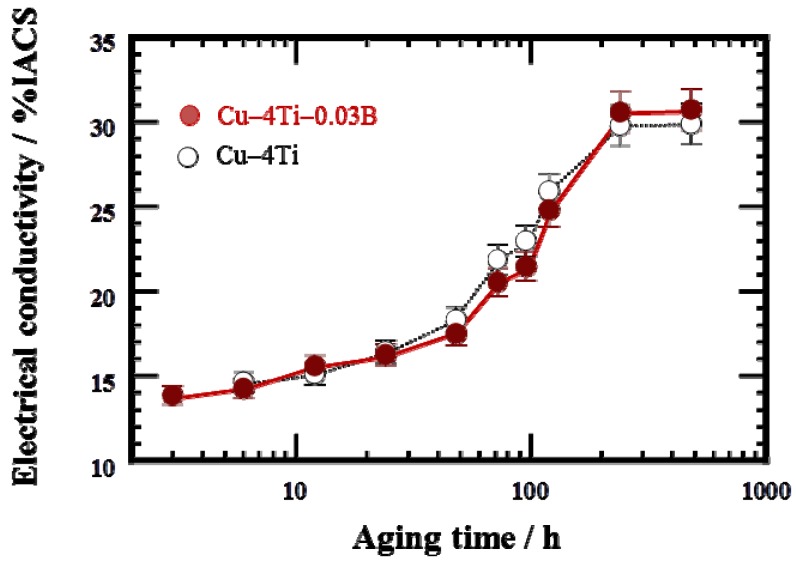
Electrical conductivity of Cu–4Ti–0.03B and Cu–4Ti alloys that were aged at 450 °C. The conductivity is expressed in % IACS, the percentile of the conductivity with respect to that of annealed pure copper.

Figure 7 shows the variation in electrical conductivity at room temperature of the Cu–4Ti–0.03B and Cu–4Ti alloys as a function of aging time at 450 °C. The electrical conductivity of both the as-quenched specimens with and without B doping was 3% IACS (% IACS is defined as the percentile based on the electrical conductivity of an international annealed copper standard (IACS) at 25 °C, 5.80 × 10^7^ Ω^–1^ m^–1^). The conductivity of the Cu–4Ti–0.03B alloy specimens increased gradually with aging and became almost constant within experimental accuracy at 30% IACS after 240 h. 

For the age-hardenable Cu–Ti alloys, the electrical conductivity at the peak-hardened state is one of the most important parameters. In the case of the Cu–4Ti alloy specimens without B doping, the electrical conductivity at a peak-hardness of 270 Hv (aging time: 12 h) was less than 15% IACS, as shown in Figure 7. On the other hand, in the case of the Cu–4Ti–0.03B alloy specimens, the aging range exhibiting peak hardness of more than 270 Hv was extended between 12 and 72 h. Eventually, the electrical conductivity at the peak hardness increased to 20% IACS in the specimens aged for 72 h. Therefore, we conclude that B doping of age-hardenable Cu–Ti alloys also improves the balance of the strength and electrical conductivity.

## 3. Experimental Section 

### 3.1. Materials and Preparation of Alloy Specimens

It has been reported that the solubility limit of B in a copper solid-solution is 0.06 (at.%) at room temperature [16,17]. In this study, in order to investigate the effect of the solid-solute B on the precipitation behavior of Cu–Ti alloys, an alloy containing a small amount of boron within the solubility limit was prepared by the following processes. An ingot with a length of 80 mm, width of 40 mm, and thickness of 10 mm was prepared by high-frequency induction melting in vacuum, using 99.99% copper, 99.99% titanium, and 99% boron strips as raw materials. The ingot was homogenized at 900 °C for 24 h, after which it was cold-rolled to a thickness of 0.6 mm. From this plate, strips measuring 60 mm in length and 5 mm in width, and pieces for tensile tests measuring 10 mm in gauge length and 2 mm in width were cut. The specimens were solution-treated at 850 °C for 6 h in vacuum and quenched in ice water. For comparison, Cu–4Ti alloy specimens without B were also prepared by the same process. The composition, analyzed chemically by ICP-AES, was Cu, 4.0 at.% Ti, and 0.032 at.% B for the alloy specimens with B doping and Cu and 3.9 at.% Ti for the alloy specimens without B; the specimens are referred to as Cu–4Ti–0.03B and Cu–4Ti alloys, respectively, for simplicity. The specimens were mechanically polished using a slurry with fine alumina particles to remove contaminants from the surface. They were then aged at 450 °C for 1–480 h under vacuum.

### 3.2. Characterization of Alloy Specimens

The microstructures of the specimens were evaluated using an FE-SEM (JSM-7001F, JEOL, Tokyo, Japan) operating at 15 kV, EBSD analysis using an SEM (JSM-5100A, JEOL, Japan) with an orientation imaging microscopy (OIM) system (INCA, Oxford Instruments, Oxford, UK), and a ToF-SIMS instrument (TOF.SIMS, ION-TOF GmbH, Münster, Germany). For the microstructural observations, the specimens were mechanically polished using 2000-grade emery paper and then electrochemically polished with a solution of 40 vol.% phosphoric acid at 0 °C, at an applied DC voltage of 1 V for 30 s. Indentations were made on the specimen by a Vickers hardness tester to observe the same region with EBSD and ToF-SIMS analysis. For the ToF-SIMS measurements, we used primary Bi_3_^2+^ ion beams with an energy of 25 keV. Since the exposure of the specimens to a low partial pressure of oxygen would enhance the intensity of the secondary ions, the specimen surfaces were exposed to oxygen gas with a partial pressure of 2 × 10^–4^ Pa. Details of the ToF-SIMS measurement procedures are described in the literature [18]. 

To characterize the composition of the age-induced precipitates, we extracted the precipitate particles formed during aging by the following chemical-dissolution technique. First, the specimens were cut into smaller pieces with dimensions smaller than 2 × 2 × 0.2 mm^3^ so that they would efficiently dissolve in the solution. These pieces were submerged in a 7 mol/L nitric acid solution at 273 K for approximately 20 min [10,15]. After the Cu solid-solution phase in the pieces had dissolved in the acidic solution, the insoluble precipitates remained. The solution was then passed through a membrane filter, and the filtered precipitates were rinsed thoroughly with pure water and dried in a desiccator. Insoluble precipitates and filtrates (in which the matrix was chemically dissolved) were separated from the specimens, and their mass fractions of elemental B and Ti were determined through an ICP-AES-based technique performed using a spectrometer (IRIS Advantage DUO, Thermo Fisher Scientific, Waltham, MA, USA) [19]. 

The electrical conductivity of the aged specimens, with a length of 60 mm, was measured at room temperature by a standard DC four-probe technique using a micro-ohm meter (34420A, Agilent, Santa Clara, CA, USA). Vickers hardness tests were conducted with an applied load of 2.94 N and a holding time of 10 s using a hardness-testing machine (MVK-E, Mitutoyo, Tokyo, Japan). The hardness number was determined by averaging the results of more than 10 tests. Tensile tests were performed using a universal tensile testing machine (UTA-100KN, A&D, Tokyo, Japan) at room temperature with a strain rate of 1.7 × 10^−4^ s^−1^. The fracture surfaces were also examined by the FE-SEM (JSM-7001F, JEOL, Tokyo, Japan).

## 4. Conclusions

We prepared Cu–4Ti (at.%) alloy specimens doped with a small amount of B and investigated their microstructure, mechanical strength, and electrical conductivity upon aging at 450 °C. The microstructural evolution of the quenched Cu–4Ti–0.03B alloys revealed that the B dopant was preferentially located at the grain boundaries of the supersaturated solid-solution phase. In the initial stage of aging for the Cu–4Ti–0.03B alloy specimens, formation of the fine metastable β′-Cu_4_Ti continuously occurred in the matrix phase grains, which was also the case for the Cu–4Ti alloy specimens without B. In a later stage of aging, the cellular discontinuous precipitates composed of the stable β-Cu_4_Ti and solid-solution laminates were formed at the grain boundaries, and they grew together with the fine β′-Cu_4_Ti precipitates. Compared with the Cu–4Ti alloy specimens without B doping, the growth of the discontinuous precipitates was suppressed in the Cu–4Ti–0.03B alloy specimens during the over-aging period of 72–120 h. This resulted in enhancement of the hardness and tensile strength in the over-aging period and eventually led to improvement in the balance of mechanical and electrical properties. It was also found that the elongation to fracture was increased in the B-doped alloy. Therefore, it can be concluded that B doping in the age-hardenable Cu–Ti alloys can improve the mechanical and electrical properties, especially in the over-aging stage. 

## References

[1] Soffa W.A., Lauglin D.E. (2004). High-strength age hardening copper–titanium alloys: Redivivus. Prog. Mater. Sci..

[2] Borchers C. (1999). Catastrophic nucleation during decomposition of Cu–0.9 at.% Ti. Philo. Mag. A.

[3] Hameda A.A., Blaz L. (1998). Microstructure of hot-deformed Cu–3.45 wt.% Ti alloy. Mater. Sci. Eng. A.

[4] Markandeya R., Nagarjuna S., Sarma D.S. (2004). Effect of prior cold work on age hardening of Cu–4Ti–1Cr alloy. Mater. Sci. Eng. A.

[5] Semboshi S., Konno T.J. (2008). Effect of aging in hydrogen atmosphere on electrical conductivity of Cu–3 at.% Ti alloy. J. Mater. Res..

[6] Semboshi S., Nishida T., Numakura H., Al-Kassab T., Kirchheim R. (2011). Effects of aging temperature on electrical conductivity and hardness of Cu–3 at. pct Ti alloy aged in a hydrogen atmosphere. Metall. Mater. Trans. A.

[7] Laughlin D.E., Cahn J.W. (1975). Spinodal decomposition in age hardening copper-titanium alloys. Acta Metall..

[8] Laughlin D.E., Cahn J.W. (1974). The crystal structure of the metastable precipitate in copper-based copper-titanium alloys. Scr. Metall..

[9] Datta A., Soffa W.A. (1976). The structure and properties of age hardened Cu-Ti alloys. Acta Metall..

[10] Semboshi S., Ishikuro M., Sato S., Wagatsuma K., Iwase A., Takasugi T. (2014). Investigation of precipitation behavior in age-hardenable Cu–Ti alloys by an extraction-based approach. Metall. Mater. Trans. A.

[11] Ecob R.C., Bee J.V., Ralph B. (1979). The structure of the β-phase in dilute copper–titanium alloys. Phys. Status Solidi.

[12] Ecob R.C., Bee J.V., Ralph B. (1980). The cellular reaction in dilute copper-titanium alloys. Metall. Trans. A.

[13] Sobhani M., Mirhabibi A., Arabi H., Brydson R.M.D. (2013). Effects of in situ formation of TiB_2_ particles on age hardening behavior of Cu–1 wt% Ti–1 wt% TiB_2_. Mater. Sci. Eng. A.

[14] Furuta R., Sato T., Kobayashi I., Tezuka Y. (2014). The effects of B addition on discontinuous precipitation of Cu-Ti alloy. J. Jpn. Inst. Copp..

[15] Semboshi S., Ishikuro M., Sato S., Wagatsuma K., Takasugi T. (2013). Extraction of precipitates from age-hardenable Cu–Ti alloys. Mater. Charact..

[16] Chakrabarti D.J., Laughlin D.E. (1994). Phase Diagrams of Binary Copper Alloys.

[17] Wang C.P., Guo S.H., Tang A.T., Pan F.S., Liu X.J., Ishida K. (2009). Thermodynamic assessments of the Cu–B and Cu–Tm systems. J. Alloys Compd..

[18] Suzuki S., Shishido R., Tanaka T., Abe F. (2014). Characterization of the inhomogeneous distribution oflight elements in ferritic heat-resistant steels by secondary ion mass spectrometry. ISIJ Int..

[19] Hosoya M., Tozawa K., Takada K. (1986). Rapid technique for distillation of methyl borate for ICP atomic-emission spectrometric determination of boron in steel. Talanta.

